# Reproductive performance of sows inseminated with semen doses stored for up to seven days in long-term extender in a field condition

**DOI:** 10.21451/1984-3143-AR2019-0121

**Published:** 2020-02-11

**Authors:** Matheus Schardong Lucca, Rafael da Rosa Ulguim, Fernando Pandolfo Bortolozzo, Ivo Wentz, Janaina Colecha Rocha, Mayara Corrêa Dias de Souza, Roberto Vasconcelos Escobar, Kérlin Calderam, Pedro Ivo de Quadros, Raquel Ausejo Marcos

**Affiliations:** 1 Departamento de Medicina Animal, Faculdade de Veterinária, Universidade Federal do Rio Grande do Sul, Porto Alegre, RS, Brasil; 2 Bretanha Importação e Exportação, Passo Fundo, RS, Brasil; 3 Magapor S.L., Zaragoza, España

**Keywords:** artificial insemination, boar stud, storage time

## Abstract

This study aimed to evaluate the reproductive performance of sows inseminated with semen doses preserved at 15-18 °C for up to seven days in long-term extender (Duragen^®^). Parity one (PO1) to PO7 sows were randomly assigned to the following groups: AI1-3 (n=190), insemination with semen doses stored between one and three days; and AI5-7 (n=124), insemination with semen doses stored between five and seven days. Sows were submitted to estrus detection twice a day. Post-cervical insemination according to weaning-to-estrus interval was performed. The farrowing rate (FR) did not differ between the groups (AI1-3=83.2%; AI5-7=82.2%; p>0.05) nor did the total number of piglets born (TPB; AI1-3=14.2±0.3; AI5-7=14.5±0.3; p>0.05). Considering the semen dose most likely responsible for fertilization according to its storage time (1, 2-3, 5, and 6-7 days), the FR (72.7%, 87.8%, 85.7%, and 79%, respectively) and TPB (14.4, 14.0, 14.9, and 13.5, respectively) were similar among the groups (p>0.05). In conclusion, the use of semen doses extended with long-term extender stored for up to seven days did not impair the reproductive performance of sows. Therefore, it’s using could optimize production efficiency and logistics of semen dose deliveries to sow farms.

## Introduction

In swine production, artificial insemination (AI) is used with liquid-stored semen. Extenders are used to increase the volume of the ejaculate, provide energy to sperm, protect against cold shock, maintain a stable pH, ensure an adequate osmotic pressure, and prevent bacterial growth during storage (Yeste, 2017). The extenders are divided into two main groups according to the capability of promoting the viability of sperm cells over time: short-term (up to three days) and long-term (more than four days) (Yeste, 2017). Despite this, usually, semen doses are used up to three days after collection in commercial conditions regardless of the extender (Pinart et al., 2015).

The use of semen doses stored for a long time could bring a reduction in the frequency of semen delivery for each farm, therefore, optimizing the production logistics of the boar studs (Anil et al., 2004). Additionally, there is little information on reproductive performance using semen doses stored for more than five days in a field condition, even when stored in long-term extenders. Therefore, this study aimed to evaluate the reproductive performance of sows inseminated with semen doses preserved up to seven days in a long-term extender.

## Materials and methods

All procedures were approved by the Ethics Committee (CEUA 34329/2018) of the Federal University of Rio Grande do Sul, Brazil.

### Animals and farms

The experiment was performed during the winter in two commercial farms (boar stud and sow farm) located in Southern Brazil (Santa Catarina State). The animals were housed with partially slatted floor, automatic feeders, and *ad libitum* access to water. The boar stud had a temperature-controlled facility (18-23 °C) and in the sow farm the curtain management was used. The weaned sows were housed in individual crates until 50 days post-insemination and then were moved to gestation pens (10 sows/pen; 2.5 m^2^/sow). Five days before the expected farrowing date, they were moved to farrowing rooms.

### Experimental design

A total of 314 multiparous weaned sows (Fertillis 25, Agroceres PIC^®^, Patos de Minas, Brazil) were randomly assigned during five weeks according to parity (one to seven), weaning-to-estrus interval (WEI), previous number of total piglets born, and lactation length (18 to 26 days) into the following groups: AI1-3: sows inseminated with semen doses stored between one and three days; and AI5-7: sows inseminated with semen doses stored between five and seven days.

### Semen processing, transport, and storage

Eight boars (Agroceres PIC^®^) were previously selected according to the ability for maintaining the total sperm motility above 70% at day 8 of storage using the Duragen^®^ extender (Magapor, Zaragoza, Spain). Boar sperm was collected weekly by the gloved hand method and immediately after the collection, sperm motility, morphology, and concentration were assessed by computerized semen analysis (Magavision^®^; Magapor, Zaragoza, Spain). Only ejaculates with a total motility ≥70% and morphological defects <20% were processed. Each ejaculate was diluted in the one-step method to obtain blisters (Smallbag, Magapor, Zaragoza, Spain) with 1.25×10^9^ viable sperm cells in 45 mL volume. Homospermic semen doses were produced from the same ejaculate (on split-sample basis); half of them were used between one and three days of storage and the other half was used between five and seven days of storage. The semen doses were transported in a thermal box and arrived at the sow farm approximately two hours after dilution and were stored at 15-18 °C. One sample from each group and boar was assessed daily for total motility under bright field microscopy (200×). Only doses with a total motility ≥70% were used for AI.

### Estrous detection and artificial insemination

Estrus detection was performed twice a day (at 7:30 am and 3:30 pm) starting at weaning by fence-line boar contact and back-pressure test. Heterospermic insemination using the post-cervical technique was performed based on the WEI according to the following protocol: one to three days of WEI, first insemination on the subsequent shift after estrus onset; four to six days of WEI, first insemination at estrus onset. Later inseminations were performed at 24 hours interval until the sow showed standing estrus. The pregnancy rate (PR), farrowing rate (FR), the total number of piglets born (TPB), number of piglets born alive (BA), number of stillborns (SB), and the number mummified (MM) were recorded.

### Ultrasonographic examination

A sub-sample of 247 sows was evaluated once a day in 24 h intervals from the onset of estrus until 24 h after the last AI to evaluate the occurrence of ovulation. The evaluation was performed by the same trained technician using a linear transrectal transducer (A6V, Sonoscape^®^ Co. Ltda, Shenzhen, China, 5.5-8 MHz). The information obtained was used to identify the most probable semen dose responsible for fertilization. The most probable semen dose was considered to be the one infused right before ovulation. Based on this response, the reproductive performance was analyzed according to the storage time most probably responsible for fertilization as follows: 1, 2-3, 5, and 6-7 days of storage prior to insemination.

### Statistical analysis

Statistical analyses were performed using *Statistical Analysis System* software (SAS, version 9.4) using the GLIMMIX procedure. The group of storage times was considered a fixed effect and the week a random effect. The frequency variables were analyzed using logistic regression in the binary distribution. Npar1way was used to analyze the percentage of stillborns and mummified. The averages were compared using the Tukey-Kramer test.

## Results

On average, 2.6±0.5 semen doses per/sow/estrus were used. Moreover, only 8.6% of the batches of semen doses produced were discarded due to low motility (<70%) for both groups. The reproductive performance was not affected (p>0.05) in sows in the group AI5-7 compared to AI1-3 (Table 1). Considering the semen dose that was most likely responsible for fertilization, the FR was not affected (p>0.05) when doses were used at 1 (72.7%; 72/99), 2-3 (87.8%; 43/49), 5 (85.7%; 48/56), or 6-7 (79.0%; 34/43) days of storage. The TPB also did not differ (p>0.05) when analyzed according to the semen dose responsible for fertilization (1 d = 14.4±3.0, 2-3 d = 14.0±3.6, 5 d= 14.9±4.2, and 6-7 d = 13.5±3.9; mean±SD). No differences in FR or TPB were observed (p>0.05) when 1 *vs* 6-7 days were analyzed separately.

**Table 1:** Reproductive performance of sows inseminated with semen doses stored for different lengths of time (mean±SEM).

**Variables**	**Semen storage time (days)**		**Probability value**
	**1-3**	**5-7**	
Number of sows	190	124	-
Weaning-to-estrus interval, d	3.8±0.06	3.9±0.07	0.37
Pregnancy rate, % (n/n)	85.8 (163/190)	85.5 (106/124)	0.94
Farrowing rate, % (n/n)	83.2 (158/190)	82.2 (102/124)	0.84
Total piglets born	14.2±0.3	14.5±0.3	0.38
Piglets born alive	13.0±0.2	13.3±0.3	0.57
Stillborns, %	6.4±0.5	6.0±0.7	0.49
Mummified, %	1.5±0.3	2.3±0.6	0.44

## Discussion

This study challenged the use of semen doses in long-term extender and found no impairment of FR and TPB when doses were used up to seven days of storage in a field condition. In studies performed in the past, the use of semen doses stored beyond three days had reduced reproductive performance, regardless of the extender used (Waberski et al., 1994). However, later experiments showed a satisfactory reproductive performance using doses stored for four to five days in long-term extender (Haugan et al., 2007), which was corroborated by the results of the present study. This better performance in relation to past studies is possibly related to the current long-term extenders containing improved buffering agents, antioxidants, and antibiotics (Knox, 2016), which might be able to maintain sperm fertility for a longer period.

Few farms have used semen doses beyond four days of storage even when diluted in long-term extenders (Knox, 2016). Aiming to improve reproductive performance, some production systems routinely use the long-term extender within two days of storage. However, no benefit has been observed compared to using short-term extenders (Pinart et al., 2015).

Even though considering the differences between estrus detection and the first AI (based on the AI protocol used) we did not observe differences (P≥0.50) for FR and TPB according to different WEI (data not shown). Furthermore, the time for insemination to obtain better reproductive performance is up to 24 h before the last insemination (Soede et al., 1995), which is in agreement with the interval used to identify the semen dose responsible for fertilization in this study. Thus, when the data were analyzed considering the semen dose most likely responsible for fertilization, the use of doses stored for 6-7 days did not affect the FR and TPB compared to the use of those stored for only one day. These data agree with a retrospective study that showed no reduction in TPB using doses stored for up to seven days in long-term extender (Anil et al., 2004). However, different from the study previously cited, in the present trial the different ages of semen doses were controlled for each sow during all inseminations. We should consider that all the boars used in our trial were previously selected according to their capability of maintaining the motility of sperm cells up to eight days of storage. Additionally, only semen doses with motility above 70% were used, since it is known that total motility below 60% has a negative impact on reproductive performance (Flowers, 1997). The long-term storage capability of semen doses might improve production efficiency and simplify logistics due to a reduction in the frequency of semen dose deliveries to sow farms (Haugan et al., 2007).

The reproductive performance was below that expected. This is probably due to the occurrence of vulvar discharge in the sows during the experiment, which is associated with a reduction in the conception rate and TPB (Dee, 1992). We do not have an explanation for the occurrence of vulvar secretion; however, it was not associated with the treatments (AI1-3=11.5% and AI5-7=9.7%; p=0,70).

## Conclusion

The use of semen doses preserved for up to seven days in long-term extender can be considered for a practical application without affecting the reproductive performance of sows.
